# The dynamics of forming a triplex in an artificial telomere inferred by DNA mechanics

**DOI:** 10.1093/nar/gkz464

**Published:** 2019-05-22

**Authors:** Ning Li, Junli Wang, Kangkang Ma, Lin Liang, Lipei Mi, Wei Huang, Xiaofeng Ma, Zeyu Wang, Wei Zheng, Linyan Xu, Jun-Hu Chen, Zhongbo Yu

**Affiliations:** 1 State Key Laboratory of Medicinal Chemical Biology, College of Pharmacy, Nankai University, Haihe Education Park, 38 Tongyan Road, Tianjin 300353, China; 2 State Key Laboratory of Precision Measuring Technology and Instruments, Tianjin University, Tianjin 300072, China; 3 National Institute of Parasitic Diseases, Chinese Center for Disease Control and Prevention, WHO Collaborating Center for Tropical Diseases, National Center for International Research on Tropical Diseases, Key Laboratory of Parasite and Vector Biology, Ministry of Health, Shanghai 200025, China

## Abstract

A telomere carrying repetitive sequences ends with a single-stranded overhang. The G-rich overhang could fold back and bind in the major groove of its upstream duplex, forming an antiparallel triplex structure. The telomeric triplex has been proposed to function in protecting chromosome ends. However, we lack strategies to mechanically probe the dynamics of a telomeric triplex. Here, we show that the topological dynamics of a telomeric triplex involves 3′ overhang binding at the ds/ssDNA junction inferred by DNA mechanics. Assisted by click chemistry and branched polymerase chain reaction, we developed a rescue-rope-strategy for mechanically manipulating an artificial telomeric DNA with a free end. Using single-molecule magnetic tweezers, we identified a rarely forming (5%) telomeric triplex which pauses at an intermediate state upon unzipping the Watson–Crick paired duplex. Our findings revealed that a mechanically stable triplex formed in a telomeric DNA can resist a force of 20 pN for a few seconds in a physiological buffer. We also demonstrated that the rescue-rope-strategy assisted mechanical manipulation can directly rupture the interactions between the third strand and its targeting duplex in a DNA triplex. Our single-molecule rescue-rope-strategy will serve as a general tool to investigate telomere dynamics and further develop triplex-based biotechnologies.

## INTRODUCTION

**Figure 1.**
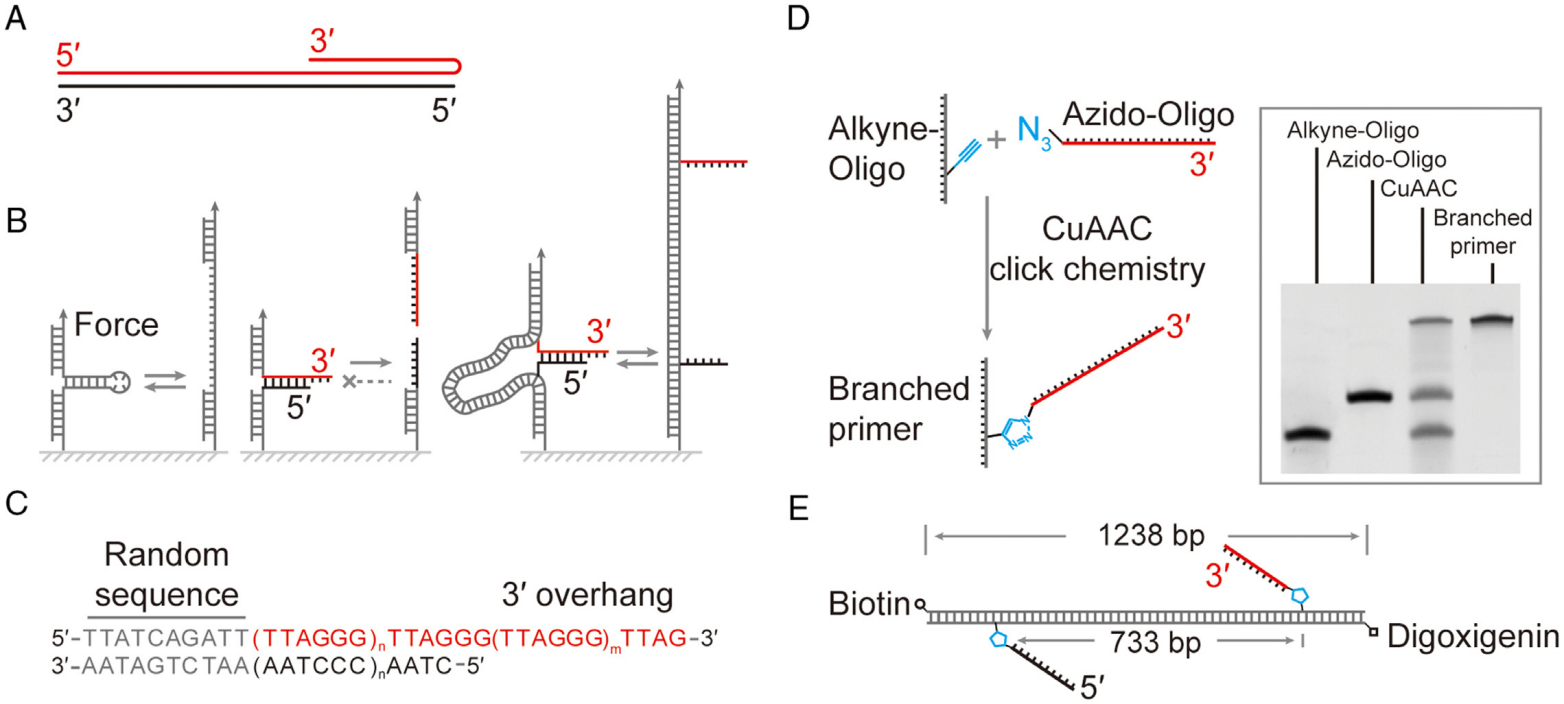
Rescue-rope-strategy for examining an artificial telomere with an open end. (**A**) Telomere DNA forms a triplex conformation. Red and black indicate the G-rich and C-rich strand, respectively. (**B**) Rescue-rope-strategy for probing a telomere with a free end. Conventional mechanical pulling assays can repetitively manipulate structures with a closed end, e.g. a DNA hairpin (left). A structure with a free and open end cannot undergo circles of mechanical pulling-relaxing (middle). A rescue-rope-strategy assisted by dsDNA allows manipulations repetitively on a structure with a free end, e.g. a telomere (right). Color coding the same as (A). (**C**) The design of an artificial telomere. A random sequence at the blunt upstream end assures the same configuration of the artificial telomere in melting/reannealing circles. The number of TTAGGG motif can vary in a duplex and 3′ overhang. Here, *n* = 5 and *m* = 2. (**D**) Click chemistry to generate a branched primer using alkyne-oligo and azido-oligo. The gel result of polyacrylamide gel electrophoresis shows the reactants, products of Cu(I)-catalyzed azide-alkyne cycloaddition (CuAAC) and branched primer carrying a telomeric G-rich strand after purification. (**E**) Final construct of an artificial telomere on a rescue-rope DNA. The telomeric G-rich (red) and C-rich (black) ssDNA are positioned at two sites 733 bp away on the rescue-rope DNA, which carries biotin and digoxigenin modifications.

DNA triplexes are biologically important for genomic instability (1), DNA repair and recombination (2–6) as well as telomere protection (7,8). Telomeric DNA consists of tandemly repeated sequence at each natural end of a eukaryotic chromosome (9,10). The repeating motif of TTAGGG runs 2–15 kilobases (kb) in the telomeric duplex in human chromosomes (11). The G-rich strand protrudes in a 5′-3′ direction beyond the double-stranded (ds) duplex to form a single-stranded (ss) overhang that is 50–300 nucleotides (nts) long (12,13). The G-rich overhang could fold back and bind in the major groove of the upstream dsDNA, forming an antiparallel triplex conformation regarding the chemically identical strand (Figure 1A) (7). The telomeric triplex has been proposed to protect the ends of a chromosome and regulate the functions of a telomere (8). The formation of a telomeric triplex should be reversible upon replication, transcription, and elongation of a telomere. Failure in telomere protective mechanisms causes telomere attrition, leading to aging and diseases (11,14–15).

Single-molecule force spectroscopy, such as AFM, optical tweezers and magnetic tweezers, has been used to study the dynamics of telomeres (12,16), especially the recognition of telomeric motifs, the assembly of shelterin on dsDNA, and the high-order organization on telomeric ssDNA (13,17–21). The conventional DNA configuration in a mechanical assay generally contains no free ends, e.g. ds/ssDNA with all the ends immobilized on surfaces, which cannot be applied on a telomeric triplex with a free overhang (Figure 1B) (20,22–24). The interactions between the third strand and the duplex in a triplex DNA have been explored using single-molecule force spectroscopy. In an AFM setup, rupture events were examined between a third strand on a tip and a duplex on a gold surface (25). Magnetic tweezers were recently employed to investigate parallel triplex formed between stretched ssDNA and its freely diffused homologous dsDNA (3). We lack mechanical strategies to probe the existence and dynamics of triplexes formed in a telomeric configuration.

Here, we developed a rescue-rope-strategy for mechanically manipulating an artificial telomeric DNA with a free end. The rescue-rope-strategy is based on click chemistry and branched polymerase chain reaction (PCR). We used AFM, magnetic tweezers and gel electrophoresis to validate the successful construction of the artificial telomeric DNA. Using single-molecule magnetic tweezers, we found that telomeric triplexes can be formed by eight repeats of TTAGGG. Upon unzipping the Watson–Crick paired duplex, the unfolding event pauses at an intermediate state which suggests the disruption of a mechanically stable triplex. By repetitively unfolding/folding telomeric triplexes, we measured the probability and characteristic time of the pauses in force-jump assays. The formation of telomeric triplexes depends on the G-rich overhang and the DNA sequence at the ds/ssDNA junction. Mutation of TTAGGG to AAAGGG dramatically increased the formation probability of telomeric triplexes. Our finding suggests that the triplex dynamics may play a role in telomere protection. We also demonstrated how to directly rupture a triplex-forming oligonucleotides (TFOs) from the major groove of a dsDNA using rescue-rope-strategy assisted mechanical manipulations. Our single-molecule rescue-rope-strategy will serve as a universal tool to investigate telomere dynamics and further develop triplex-based biotechnologies, such as targeting virus genes and delivering drugs (26–30).

## MATERIALS AND METHODS

All the modified oligos underwent HPLC purification and ESI-MS characterization. Other than specifically noted, we have purchased all the restriction enzymes and ligases from the New England Biolabs (Ipswich, USA).

### Preparation of rescue-rope construct with branched telomeric oligos

We purchased azide and alkyne modified oligos (Supplementary Table S1) from IDT Inc. (Skokie, USA) or Sangon Biotech (Shanghai, China). To set up the click chemistry reaction of Cu(I)-catalyzed azide-alkyne cycloaddition (31,32), we mixed 5 μl of TBTA at 100 mM and 2.5 μl of CuSO_4_ at 50 mM in phosphate-buffered saline buffer (pH 7.4). We then supplied the mixture above with 6.2 μl of sodium ascorbate at 160 mM. After 10 min at room temperature avoiding light, we added 5 μl each of azide and alkyne modified oligos at 0.5 mM, reaching a final volume of 25 μl. Shaking at 200 rpm at 30°C for 1 h, we supplied the reaction with another 5 μl of TBTA (100 mM), 2.5 μl of CuSO_4_ (50 mM) and 6.2 μl of sodium ascorbate (160 mM). The reaction ran for an additional 3 h. We purified the products of branched oligos using 15% polyacrylamide gel electrophoresis with 7 M urea.

The triazole connection, formed in the branched oligos after click chemistry, is thermally and hydrolytically stable. To construct rescue-rope DNA we used the branched oligos as primers in PCR. We purchased from BGI (Beijing, China) the biotin and digoxigenin modified primers which pair with branched oligos for PCR amplification (Supplementary Table S1). To avoid interactions between rescue-rope DNA and telomeric sequences, we chose a fragment of lambda DNA as a PCR template which contains only one trinucleotide of CCC/GGG in 1238 bp. We used Kodaq 2X PCR MasterMix (ABM) or PrimeSTAR GXL DNA Polymerase (Takara) to run the experiment of branched PCR.

We next used the conventional digest/ligation strategy with the restriction enzymes of BbvcI and BssSaI, as well as the T4 DNA ligase for ligation, to make the final rescue-rope construct.

### AFM

We used a commercial AFM of Multimode 8 by Brucker (Billerica, USA) and ran the measurements at room temperature of 23°C. We prepared 0.8 ng/μl DNA sample in an AFM imaging buffer containing 10 mM HEPES (pH 7.6), 4 mM MgCl_2_ and 2 mM NiCl_2_. We took 10 μl of DNA and directly loaded to the center of a freshly cleaved mica substrate. We waited 10 min for the DNA to settle down on the mica surface. We next rinsed the DNA sample using imaging buffer. We then collected and analyzed AFM images in the software coming together with the instrument.

### Single molecular magnetic tweezer

Our homemade magnetic tweezers, similar to the one previously described (33), were equipped with a 222.6 × magnification using an oil immersion objective (UPLFLN 100 × O2, Numerical aperture (N.A.) = 1.25; Olympus, Tokyo, Japan) and a CMOS camera (MC1362, Mikrotron, Germany). The gap between a pair of vertically aligned magnets is 1 mm. With M270 bead (#65305, Invitrogen), we typically used 1 pN/s or 4 pN/s as the force loading rate in force ramp experiments. The setup employed an LED for illumination in transmission, a translate stage (Physik Instrumente, M-404.1PD, Karlsruhe, Germany) to control the magnets in the vertical direction, and a rotary motor (Physik Instrumente, C-150) to rotate the magnets. A piezo nanopositioner (P-726.1CD, Physik Instrumente) can move the inverted objective in the z-direction. We made the flow cell with a single channel by a shaped parafilm which was sandwiched between two coverslips (Menzel-Gläser, 24 × 60 mm, #1, Braunschweig, Germany). The thickness of one glass coverslip and the parafilm spacer is 0.4 mm, which is inaccessible from the magnets to the beads inside the channel. We used a peristaltic pump (ISM832C, Ismatec, Wertheim, Germany) to exchange buffer. Motor control and data collection were performed in a custom-written Labview 2016 software, similar to that published in literature (34).

All the experiments using single-molecule magnetic tweezers were performed at room temperature of 23°C. We examined the telomeric construct and its mutants (except the AA mutant) in a buffer containing 20 mM HEPES (pH 7.5), 0.00315% Tween-20(v/v), 0.1 mM EDTA and 100 mM monovalent salt (LiCl, NaCl or KCl). We examined the AA mutant and the triplex formed by GA TFOs in a buffer of 10 mM Tris (pH 7.5), 30 mM NaCl, 10 mM MgCl_2_ and 0.00315% Tween 20. The buffer with specific salt was noted in figure captions.

### Gel shift assay

We heated the purified rescue-rope construct to 72°C for 3 min, then cooled the sample down by 0.5°C per minute until 23°C. Agarose gel (1.5%) was run in Tris-acetate-EDTA buffer under voltages of 8 V/cm. We used ImageJ for gel image analysis (35).

### Data treatment and analysis

Other than explicitly stated, we analyzed data of single-molecule magnetic tweezers in Matlab 2017b (MathWorks, USA). We used modified Marko-Siggia Worm-Like-Chain (WLC) model (36,37) to examine the relationship between force (F) and extension (x) of DNA,

(1) \begin{equation*}F\ = \left( {\frac{{{k_B}T}}{{{L_p}}}} \right)\left[\frac{1}{{4{{\left( {1 - \frac{x}{{{L_0}}} + \frac{F}{S}} \right)}^2}}} - \frac{1}{4} + \frac{x}{{{L_0}}} - \frac{F}{S}\right]\end{equation*}

where L_p_ is the persistence length, L_0_ is the contour length, S is the stretch modulus, *k_B_*T stands for that Boltzmann's constant times temperature.

## RESULTS

### Rescue-rope-strategy for probing an artificial telomeric DNA

We designed an artificial telomeric DNA used in single-molecule pulling assays for mechanically probing the organization of the freely open end. Our artificial telomere begins with a stretch of non-telomeric random sequence at the blunt and fixed end, continues with a duplex of TTAGGG tandem repeats and terminates in a 3′ overhang (Figure 1C). The non-telomeric random sequence helps to avoid a shift in base pairing of TTAGGG repeats in repetitive unzipping/zipping cycles. We generated a dsDNA free of (GG)_n_ motifs (*n* ≥ 1), which serves as a rescue-rope to fix the blunt end of the telomeric DNA. To probe the dynamics and mechanics of the telomeric open DNA, we constructed the artificial telomere with the duplex and 3′ overhang containing five and three repeats of TTAGGG motifs, respectively (Supplementary Table S2). The number of TTAGGG motifs could be extended for both duplex and 3′ overhang to study telomeric organization. We used magnetic tweezers to unzip the telomeric DNA from the fixed blunt end towards the open end of 3′ overhang, resulting in two individual ssDNA.

A rescue-rope-strategy assures the repetitive melting/reannealing of a telomeric DNA in mechanical unzipping/zipping assays. Both the 5′ terminus of the telomeric G-rich strand and the 3′ terminus of the complementary C-rich strand carry an azide group. The two of telomeric ssDNA branch out from a λ DNA at alkyne modified bases through Cu(I)-catalyzed azide-alkyne cycloaddition (CuAAC, Figure 1D and E). Upon melting, the click chemistry linkage by CuAAC prevents the diffusion of the unzipped telomeric ssDNA from the λ DNA. Upon reannealing, the unzipped G/C-rich ssDNA pair again in a small space confined by the λ DNA which serves as a rescue rope. We build the rescue-rope construct carrying telomeric DNA using branched primers. The two alkyne modified bases are 733 bp apart on the λ DNA rope, which is longer than the fully melted telomeric ssDNA, ∼64 nts.

### Validation of the rescue-rope DNA construct

**Figure 2.**
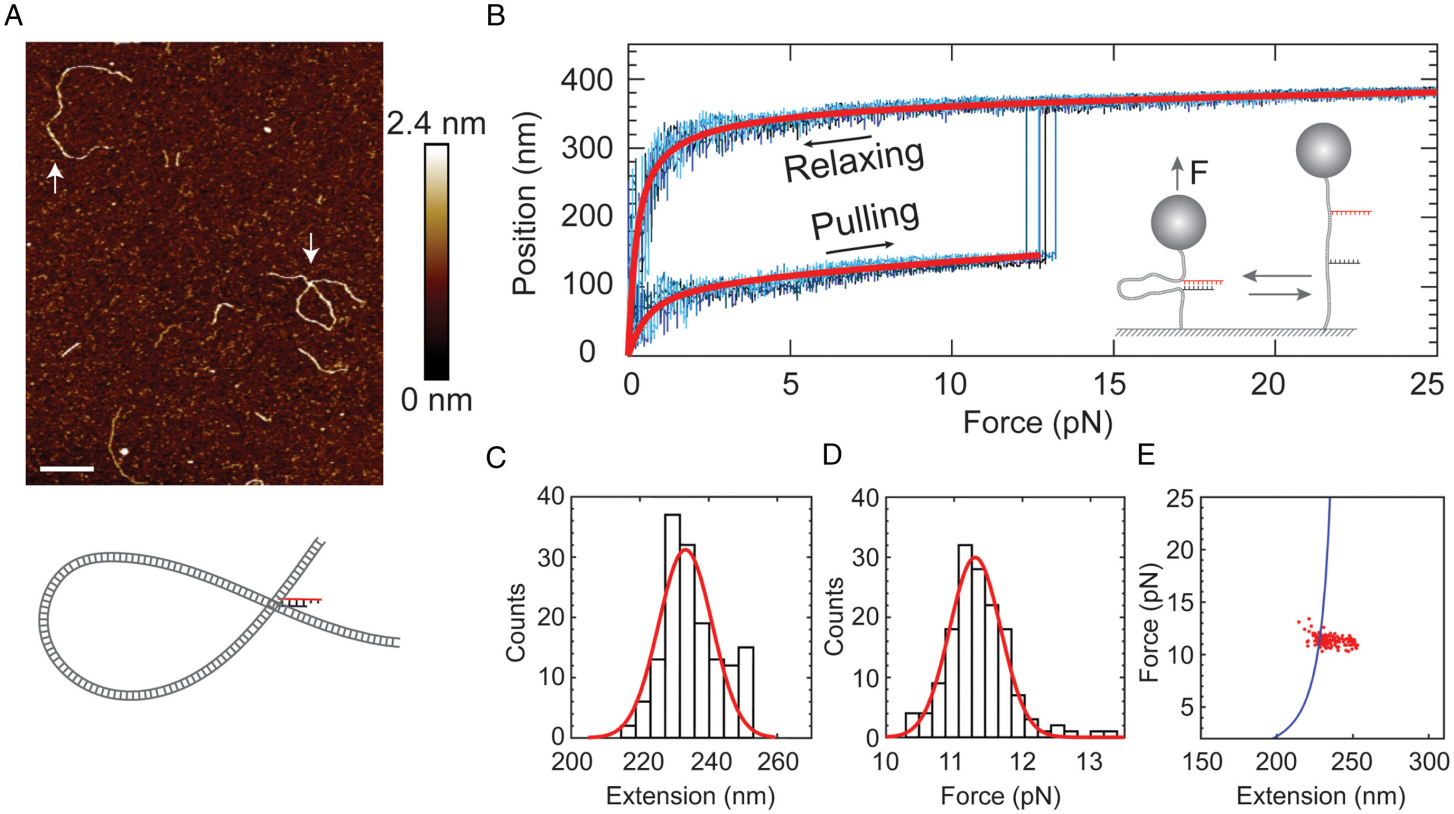
Rescue-rope-strategy validated by AFM and single-molecule magnetic tweezers. (**A**) AFM imaging of the rescue-rope DNA construct. The artificial telomere ties the rescue rope into an α-shaped conformation. The up arrow and down arrow point to the linear rescue-rope DNA and α-shaped DNA knot, respectively. The bar is 100 nm. The color bar indicates height values. The cartoon illustrates the α-shaped rescue-rope DNA in gray and the telomeric DNA in black and red. AFM imaging was performed at 23°C in a buffer containing 10 mM HEPES (pH 7.6), 4 mM MgCl_2_ and 2 mM NiCl_2_. (**B**) Mechanical manipulation of α-shaped rescue-rope DNA on single-molecule magnetic tweezers. Bead positions indicate that the DNA extensions grow as a function of forces, which follows the WLC model (red line). Four curves are repetitive trajectories of pulling-relaxing from the same molecule. The sudden leaps of bead positions suggest the unfolding of an α-shaped conformation. The cartoon illustrates the setup of a mechanical pulling-relaxing assay. The pulling assays were performed at 23°C in a buffer containing 20 mM HEPES (pH 7.5), 0.00315% Tween-20(v/v), 0.1 mM EDTA and 100 mM LiCl. (**C** and **D**) Histogram of unfolding forces (C) or the changes in the extensions (D) upon sudden leaps from the same molecule. Red line indicates a Gaussian fit. The experimental conditions were the same as that in (B). (**E**) A plot showing force versus changes in extension. Red dots are the same data as that in (C and D). The solid blue line was estimated from a WLC model with parameters from (B).

We used AFM to scan the rescue-rope DNA construct in a HEPES buffer with MgCl_2_ and NiCl_2_ on a freshly cleaved mica. We found the DNA construct carrying an artificial telomere in either a linear (*n* = 14) or an α-shaped conformation (*n* = 3) (Figure 2A). We rationalized that the α-shaped conformation is a result of a base-pairing between the G-rich and C-rich telomeric ssDNA 733 bp apart on the rescue-rope construct (Figure 2A, cartoon), and doesn’t depend on the 3′ overhang. We further deleted the single-stranded 3′ overhang in the artificial telomere to examine the conformations formed by the rescue-rope DNA construct. AFM again revealed both linear ones (76.3%) and α-shaped ones (23.7%, *n* = 130) (Supplementary Figure S1a). The telomeric DNA is situated at the cross point of the α-shaped rescue-rope and cannot be resolved in the AFM (Figure 2A). The α-shaped and linear DNA showed close contour length, 422 ± 47 nm (mean ± sd, *n* = 30) versus 407 ± 43 nm (mean ± sd, *n* = 100), which is almost identical to the full length of the rescue-rope construct, 420 nm for 1238 bp (Supplementary Figure S1b, Blue and black). The contour length of the circular part in the α-shaped DNA, 248 ± 26 nm (mean ± sd, *n* = 30), is equal to the rescue-rope DNA between the two alkyne modified sites, 249 nm for 733 bp (Supplementary Figure S1b, Red). The AFM imaging thus confirms the construction of the artificial telomeric DNA.

We further validated the rescue-rope construct using single-molecule magnetic tweezers. The two ends of the rescue-rope DNA carry biotin and digoxigenin, respectively (Figure 1E). Affinity interactions immobilize a rescue-rope DNA between a magnetic bead coated with streptavidin and a glass slide covered by the anti-digoxigenin antibody (Figure 2B, cartoon). A pair of permanent magnets provides a magnetic field which manipulates the bead on the z-direction, applying forces to the rescue-rope DNA. At a typical force loading rate of ± 4 pN/s, we repetitively stretched and relaxed the rescue-rope DNA carrying the artificial telomere between 0 and 25 pN. For the same molecule as an example (Figure 2B–E), the DNA extension smoothly increases until a sudden leap of 233 ± 11 nm at 11.3 ± 0.5 pN, then continues to extend upon stretching. During relaxing, the extension decreases in a trajectory different from that upon pulling. We applied WLC model to fit the two trajectories (Figure 2B, red), obtaining the contour length difference to be 215 ± 38 nm (*n* = 13). The dimensional difference of two trajectories measured by both the leap signal and WLC fit matches with the distance between the branching sites of telomeric oligos on the rescue-rope DNA, 249 nm for 733 bp (Figure 1E). Using the same force-ramp strategy as above on magnetic tweezers, we examined the rescue-rope DNA carrying a telomeric duplex without 3′ overhang and found similar results (Supplementary Figures S1c–f). The abrupt leap signal thus indicates that the unzipping of a telomeric duplex releases the circular part of the α-shaped rescue-rope DNA (Figure 2A, cartoon). For a rescue-rope, we can repeat the stretching-relaxing manipulation to unzip/reanneal a telomeric DNA until the bead is lost (150 times in Figure 2B–E). Similar rope rescue techniques were used in centrifuge force microscopy and AFM pulling assays (38,39). While in preparation of this manuscript, T. R. Strick demonstrated a similar strategy to investigate DNA repair using magnetic tweezers (40). The mechanical manipulation corroborates the AFM results of a successful rescue-rope construction.

Because linear DNA migrates faster than nicked circular ones in a gel shift assay (41), we ran agarose gel electrophoresis to examine the rescue-rope construction. We made a telomeric construct with the 3′ overhang permutated in which the formation of the telomeric duplex would result in an α-shaped conformation. We also made a linear dsDNA of 1238 bp containing no telomeric ssDNA, which is the same length as that of the rescue-rope dsDNA handle (Lane C_0_ in Supplementary Figure S1g). After treatment of heating and cooling of the purified rescue-rope construct, the gel electrophoresis showed multiple bands (Supplementary Figure S1g, ‘Materials and Methods’ section). Band 1 in lane C came as contamination of an intermediate product through the purification step, which is shorter than 1238 bp (Lane C_0_ versus band 1 in lane C, Supplementary Figure S1g). Band 2 in lane C migrated a bit slower than dsDNA of 1238 bp. We assigned band 2 to be the rescue-rope construct in linear conformation because two telomeric ssDNA branching out from the rescue-rope handle made the molecular weight heavier than that of 1238 bp dsDNA. Above band 2, there immediately was band 3 which we assigned to be the construct in an α-shaped conformation (Supplementary Figure S1g, lane C). The α-shaped rescue-rope construct in circular topology migrated slower than that in linear conformation. Comparing the gray intensities, we found that the ratio of band 2 over band 3 was 0.17. Band 4 and 5 showed about twice the respective sizes as that of band 2 and 3, which probably were aggregates between intermolecular interactions due to the branched telomeric ssDNA (Supplementary Figure S1g). The gel shift assay thus showed consistent results with that by AFM and single-molecule magnetic tweezers, confirming the success of the rescue-rope construction.

### Triplex formation at a telomeric end revealed by rescue-rope-strategy

**Figure 3.**
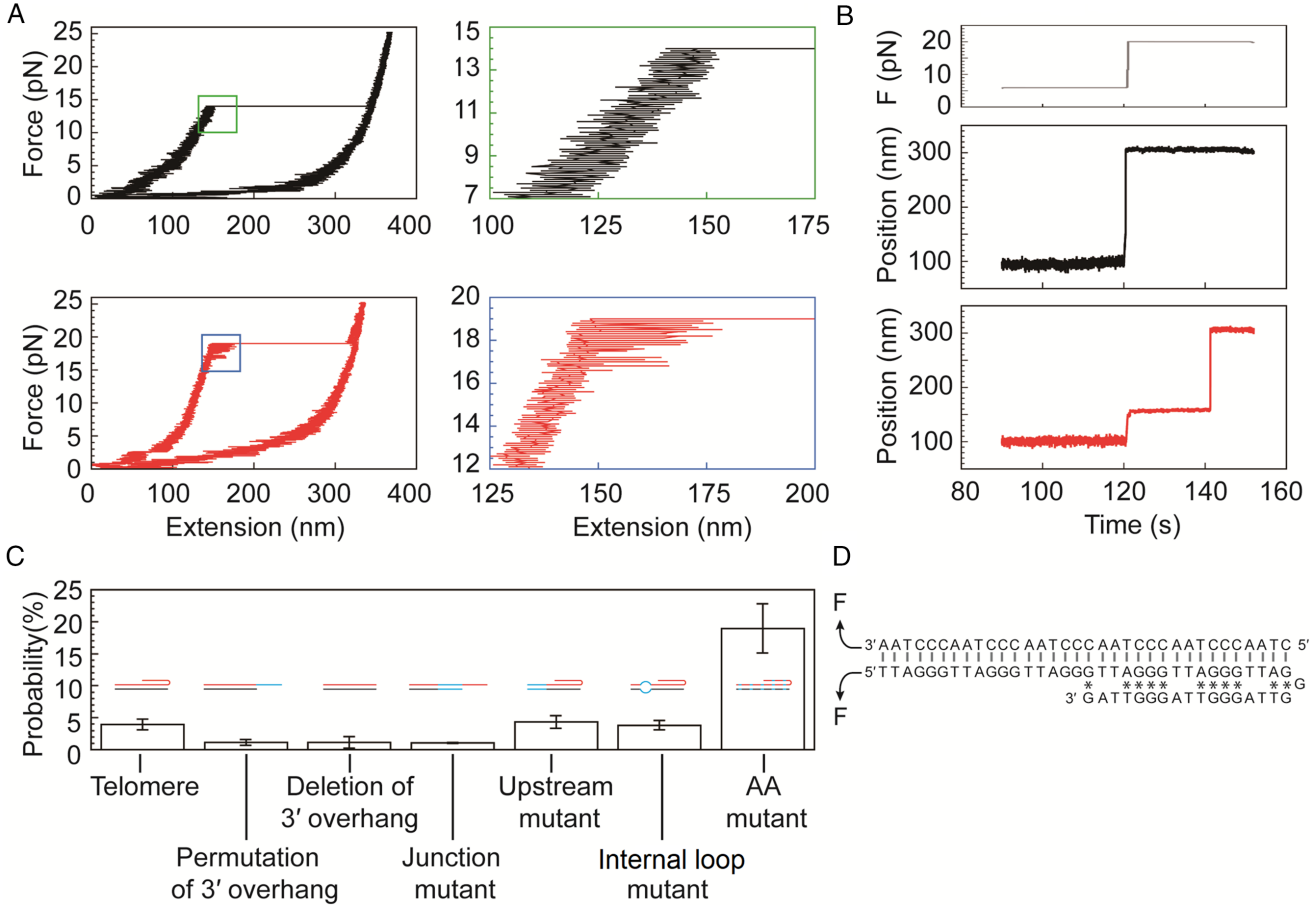
Probing telomeric triplex structures using single-molecule magnetic tweezers based on the rescue-rope strategy. (**A**) The existence of a telomeric triplex indicated by a hopping signal upon stretching a rescue-rope DNA. In the top panels, the left black trace shows an unfolding event without hopping (Zoom in for details in the right green-framed panel). In the bottom panels, the left red trace shows an infrequent hopping event (4.2% in 788 pulling-relaxing circles, zoom in for details in the right blue-framed panel). The assays were performed at 23°C in a buffer of 20 mM HEPES (pH 7.5), 0.00315% Tween-20(v/v), 0.1 mM EDTA and 100 mM NaCl. (**B**) Telomeric triplex detection in force-jump assays. The profile of manipulating forces is shown in the top panel. Rescue-rope construct undergoes a sharp transition during a force-jump from 6 to 20 pN (middle panel). Force-jump manipulations capture a rare event of unfolding a triplex, indicated by the pause as an intermediate state (bottom panel). Same buffer and temperature conditions as that in (A). (**C**) Probabilities of pausing events measured for the telomere and its mutants in force-jump assays. The telomere construct has eight repeats of TTAGGG. The mutant of permutation of 3′ overhang indicates that the bases in ssDNA tail are permutated. The mutant of deletion of 3′ overhang has the ssDNA tail deleted. The junction mutant stands for that the fifth and sixth repeats of TTAGGG are mutated at the ds/ssDNA junction. The upstream mutant represents that the first two repeats of TTAGGG are mutated which are far from the ds/ssDNA junction. In the internal loop mutant, the second TTAGGG is mutated to be mismatched as ATGTAG/ACCCTA. AA mutant changed the motif of TTAGGG to AAAGGG. Blue lines indicate mutated bases in cartoons. The buffer and temperature conditions were the same as that in (A) except that AA mutant was examined in a buffer containing 10 mM Tris (pH 7.5), 30 mM NaCl, 10 mM MgCl_2_ and 0.00315% Tween 20. (**D**) A possible triplex in an antiparallel conformation formed by our telomeric DNA. Vertical lines represent Watson–Crick pairs. Asterisks stand for reverse Hoogsteen pairs.

Upon unzipping an artificial telomere, the histogram of unfolding force revealed a single peak centered at 13 ± 2 pN in force-ramp assays when the force loading rate is ± 1 pN/s, identical to that unzipping a telomeric duplex without 3′ overhang (Supplementary Figure S2a–c versus S2d–f). When stretching a rescue-rope DNA at a time interval of 1–5 min between two subsequent assays, we occasionally (4.2%, *n* = 788) observed an abrupt leap preceded by a hopping feature, indicating that the existence of a mechanically stable structure may interrupt the unzipping of a telomeric dsDNA (Figure 3A). When a hopping feature happened, we measured unfolding forces at the final transition, i.e. the highest unfolding force, for any α-shaped construct stretched to be straight. However, neither unfolding forces nor the changes in the extension when stretching the rescue-rope construct are sensitive for examining the rarely formed structure (Supplementary Figure S2).

We further probed the stable structure using force-jump assays. To the rescue-rope setup, we quickly move magnets, increasing forces from 6 to 20 pN in 362 ± 94 ms (mean ± sd, *n* = 16) (Figure 3B, top). The typical time interval was 1–5 min between two subsequent assays. The 20 pN is stronger than the force required to unzip a telomeric dsDNA, ∼13 pN. The force-jump pulls the α-shaped rescue-rope straight, releasing the circular part locked by a telomeric DNA, as indicated by a bead displacement of 255 ± 18 nm (mean ± sd, *n* = 32) (Figure 3B, middle). Strikingly, we found an intermediate pausing state of bead displacement upon force-jump (Figure 3B, bottom). We rationalized that the unfolding of the mechanical stable structure causes the pausing signal.

We next examined the nature of the mechanical stable structure occasionally formed at the telomeric end. The averaged pausing time of the mechanical stable structure is 3 ± 2 s (mean ± sme, *n* = 8) at a testing force of 20 pN. A proposed triplex model at a telomeric end can explain our finding of the mechanical stable structure (7). In the triplex model, the purine-rich 3′ overhang folds back to bind the homologous dsDNA at the ds/ssDNA junction, forming a pyrimidine-purine-purine triplex. We permutated the 3′ overhang which reduced the pausing probability from 4% ± 1% (mean ± sd, *n* = 204) to 1.1% ± 0.5% (mean ± sd, *n* = 206) for the artificial telomere (Figure 3C). The pausing signal also vanished upon deletion of the 3′ overhang (1.1% ± 0.9%, mean ± sd, *n* = 82) (Figure 3C and Supplementary Table S2). We mutated the fifth and sixth repeats of TTAGGG in the duplex at the ds/ssDNA junction in which the pausing probability also decreased (1.0% ± 0.1%, mean ± sd, *n* = 292) (Junction mutant, Figure 3C and Supplementary Table S2). Mutation of the first two TTAGGG repeats in the upstream far from the ds/ssDNA junction didn’t change the pausing probability (4% ± 1%, mean ± sd, *n* = 278) (Upstream mutant, Figure 3C and Supplementary Table S2). We made an internal loop mutant in which the second TTAGGG motif in the telomeric duplex mismatches as ATGTAG/ACCCTA (Figure 3C and Supplementary Table S2). The pausing probability of the internal loop mutant (4% ± 1%, mean ± sd, *n* = 183) is the same as that of a normal telomeric DNA construct (Figure 3C), indicating that the position of the internal loop mutation is beyond the reach of 3′ overhang and cannot facilitate a strand invasion due to the energetic cost of bending a short dsDNA. Because the 3′ overhang and the ds/ssDNA junction are essential for the pauses upon unzipping a telomeric DNA, we assigned the pausing signal to be unfolding of a telomeric triplex in which the 3′ overhang folds back to bind the duplex at the ds/ssDNA junction (Figure 3D).

The triads of CG*G and TA*T strengthen the interactions between the 3′ overhang and the duplex by reverse Hoogsteen pairs (*) in the telomeric triplex. However, DNA triplex prefers to form in homopurine-homopyrimidine sequences. Two thymines interrupt the purine stretches in the telomeric G-rich strand while two adenines break the continuity of pyrimidine sequence in the C-rich strand (Figure 3D), explaining why the forming probability of a telomeric triplex is rare. We then mutated the telomeric motif of TTAGGG/CCCTAA to be AAAGGG/CCCTTT, called AA mutant, which makes the sequences to be homopurine-homopyrimidine (Supplementary Table S2). We examined the AA mutant using force-jump assays at a testing force of 20 pN in a buffer of 10 mM of Tris (pH 7.5), 30 mM of NaCl and 10 mM of MgCl_2_. The testing force of 20 pN is higher than the critical unfolding force of 13 ± 1 pN (mean ± sd, *n* = 8) measured for the AA mutant in force-ramp assays (force loading rate = ± 4 pN/s). We added Mg^2+^ in the buffer to stabilize the DNA triplex structure (42). We found the pausing probability to be 19% ± 4% (mean ± sd, *n* = 63) for the AA mutant which is approximately five times higher than that of the telomeric construct. For the telomeric duplex construct without 3′ overhang in the Mg^2+^ buffer, we found the pausing signal only once in 55 force-jump assays, ∼2%, ruling out the possibility that the high pausing probability for the AA mutant comes from the Mg^2+^ effect on dsDNA. We rationalized that, in the AA mutant, the 3′ homopurine overhang folds back and occupies the major groove of the duplex, forming the triads of CG*G and TA*A by reverse Hoogsteen pairs between the two purine strands. The fact that the triplex forming probability of the AA mutant is higher than that of the telomeric construct, points to that the discontinuities of purine/pyrimidine stretches in the telomeric TTAGGG/CCCTAA motifs discourage the triplex formation.

### Mechanical manipulation of a DNA triplex by rescue-rope-strategy

**Figure 4.**
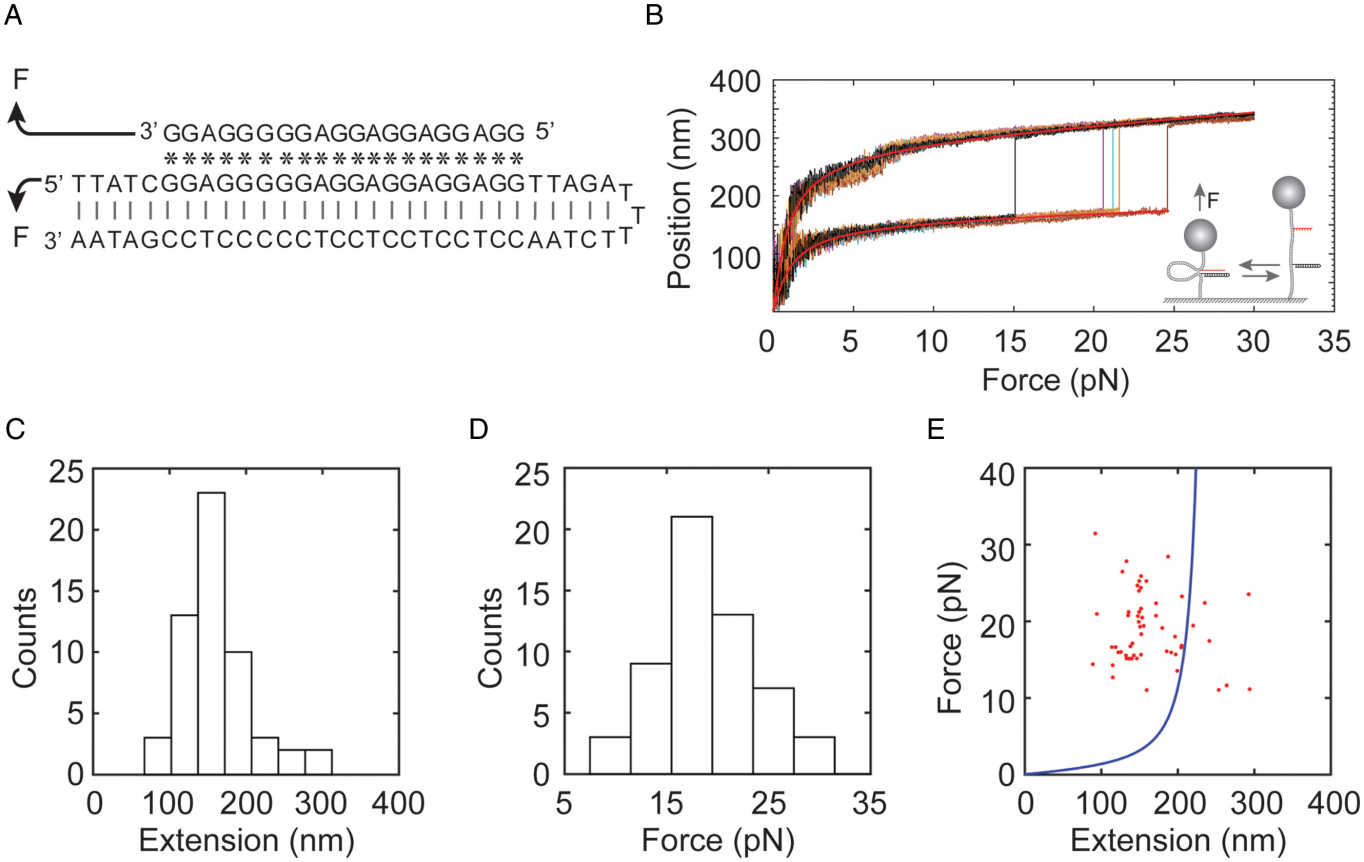
Mechanical rupture a triplex by breaking reverse Hoogsteen pairs between a TFOs and its targeting dsDNA using rescue-rope strategy. (**A**) The GA TFOs binds its targeting Watson–Crick (|) duplex by reverse Hoogsteen pairing (*), forming an antiparallel triplex regarding the purine strand. Mechanical manipulation indicated by arrows can directly disturb reverse Hoogsteen pairs in the triplex. (**B**) Force-extension curves from a single molecule showed rupture events, indicating the dissociation of the GA TFOs from a triplex structure. Smooth red lines represent the fit of a WLC model. Force-ramp assays were performed at room temperature of 23°C in a buffer containing 10 mM of Tris (pH 7.5), 30 mM of NaCl and 10 mM of MgCl_2_. (**C**–**E**). Distributions of extension (C) and force (D) as well as the plot of force versus extension (E). The solid blue line in (E) was estimated from a WLC model with parameters from (B). Same buffer and temperature conditions as that in (B).

As a proof of concept, we showed that the rescue-rope-strategy assisted single-molecule force spectroscopy can not only unzip the Watson-Crick paired dsDNA to probe DNA triplexes, but also directly disturb reverse Hoogsteen pairs between the TFOs and the purine strand of the duplex. We made a rescue-rope construct to probe the interactions between a 20-mer of TFOs and a dsDNA (Figure 4A). The 20-mer of TFOs has a homopurine sequence of 5′-GGA GGA GGA GGA GGG GGA GG-3′, called GA TFOs, which can form an extremely stable triplex with its targeting duplex as evident in literature by AFM, DMS footprint and UV melting among other methods (Supplementary Table S2) (43,44). The targeting duplex by GA TFOs was in a hairpin form with a loop of 3 Ts. We conjugated both the GA TFOs and its targeting hairpin to the rescue-rope DNA by click chemistry. In such a way, force can directly manipulate the interactions between GA TFOs and its chemically homologous strand of the hairpin (Figure 4A).

In force-ramp assays at a force loading rate of ± 4 pN/s, we mechanically probed the triplex formation in a buffer containing 10 mM of Tris (pH 7.5), 30 mM of NaCl and 10 mM of MgCl_2_ (Figure 4B). The large and sudden leaps in the force-extension curves indicate the rupture events of reverse Hoogsteen pairs formed by CG*G and TA*A, causing dissociation of the GA TFOs from the major groove of its targeting duplex. The distributions showed extension and rupture force at 164 ± 46 nm and 19 ± 4 pN (mean ± sd, *n* = 56), respectively (Figure 4C–E). The averaged change in extension was shorter than that in a monovalent buffer, which may be due to the effect of divalent cations on DNA (45–49). At the time interval of 1–5 min between two subsequent force-ramp assays, we observed the probability of rupture events to be 36% ± 2% (mean ± sd, 164 force-extension curves in total). We thus successfully demonstrated that the rescue-rope-strategy can conveniently examine the mechanics of DNA triplexes in variable force manipulating geometries.

## DISCUSSION

We developed a rescue-rope-strategy for single-molecule force spectroscopy which allowed us to repetitively manipulate structures formed at free ends of DNA, e.g. triplex DNA, telomeric DNA or broken dsDNA. After conjugation by click chemistry, DNA of interest branched out from the rope DNA handle. AFM imaging showed an α-shaped conformation adopted by the rescue-rope DNA where DNA of interest forms structures at the crossing point. Single-molecule magnetic tweezers further validated that the DNA of interest can repetitively form structures after rupture by forces. We further demonstrated how to apply the rescue-rope-strategy to mechanically probe DNA triplexes.

DNA triplexes may be in either YR*Y or YR*R (Y for pyrimidine and R for purine) configurations where the TFOs binds a purine strand of the dsDNA by Hoogsteen or reverse Hoogsteen pairs (*), respectively. The mutual direction of the chemically homologous strands could be either parallel or antiparallel in YR*Y or YR*R triplexes (50–53). A telomeric triplex model in a YR*R configuration has long been proposed to be against digestion and recombination. A DNA triplex forms in a tiny *Tetrahymena* telomere with a dsDNA of (TTGGGG)_3_ and a 3′ overhang of (TTGGGG)_2_, where the G-rich overhang folds back and occupies the major groove of the duplex by forming the CG*G triads in an antiparallel YR*R configuration (7).

Using rescue-rope-strategy assisted single-molecule force spectroscopy, we investigated the triplex formation by a short human telomeric DNA containing eight repeats of TTAGGG in the dsDNA and the ssDNA overhang (Figure 3D). The results revealed that mechanically stable triplexes occasionally form by the 3′ overhang of TTAGGG folding back to bind dsDNA, most likely through CG*G and TA*T triads. Because of the repeating motif of TTAGGG, the interactions between the 3′ overhang and the dsDNA could vary, producing a folding turn in various sizes. The folding turn in a DNA triplex could be as tight as that contains no nucleotides (54). Without forming a triplex, the telomeric duplex with repeating motif cooperatively unfolds due to forces. However, at the presence of a stabilizing triplex, the remaining duplex region (∼17 bp) could unfold before the triplex, resulting a hopping feature in a force-ramp assay or a pausing signal in a force-jump experiment. Besides pausing signals, we also observed hopping signals for all the mutants (Supplementary Figure S3). The folding back triplex structures of a telomere sequence can remain intact for an averaged time of 3 seconds at a force of 20 pN. The strong mechanical stability and long duration indicate that telomeric triplexes may get in the way of DNA cleavage and recombination, as well as force-regulated helicases (55,56) and force-sensitive RNA polymerases (57).

The human telomeric triplex forming probability probed by the force-jump assays is as low as ∼4%. One reason is that the incoherence of purine/pyrimidine stretches in the telomeric TTAGGG/CCCTAA motifs inhibits the triplex formation. Triplexes prefer homopurine-homopyrimidine DNA sequences, but can tolerate each mismatched triad at the cost energies of 3–6 kcal/mol (5–10 *k_B_*T at 25°C) (51,58–59). Two thymines thus dramatically weaken the human telomeric triplex, which is also supported by the results that AA mutant of TTAGGG to AAAGGG increased the triplex forming probability five times higher in force-jump assays. Our results on GA TFOs showed even higher triplex forming probability than that on AA mutant, suggesting that DNA sequences substantially contribute to the forming probability of triplex structures.

The second reason why we observed low forming probability of human telomeric triplex is that cations affect the stability of YR*R triplexes (42,60–62). Divalent cations coordinate to the N7 of purines and differentially stabilize YR*R triplexes by unequally enhancing the hydrogen bonds of Hoogsteen pairs (61,63). An YR*R triplex with arbitrary sequences forms in a buffer containing Zn^2+^ or Mn^2+^, but not Mg^2+^ (42). In addition, Mg^2+^ destabilizes that of (TC)_n_(GA)_n_*(AG)_n_, but stabilizes the DNA triplex of C_n_G_n_*G_n_ (42). The stabilizing effect from Mg^2+^ is so strong that the C_n_G_n_*G_n_ triplex can tolerate the disturbance of two thymines in the *Tetrahymena* telomere with TTGGGG motifs (7). We have examined the human telomeric triplex formation at a physiological pH in a buffer containing only monovalent salt but no divalent cations. We tested the human telomeric triplex formation in the presence of 10 mM Mg^2+^. However, we observed the triplex signal only once out of 55 force-jump experiments which didn’t give a higher forming probability than that in monovalent cation buffer. Because triplex formation depends on both DNA sequences and types of divalent cations, a screening of metal salts may be necessary to find which divalent cations can promote the triplex forming probability in a human telomere.

The detection method of force-jump assays may underestimate the forming probability of human telomeric triplexes. The time intervals of our force-jump assays were typically in a scale of a few minutes when forces were at zero to allow triplex formation. DNA triplex formation may not reach equilibrium in such short incubation time. A few hours of incubation time have been used in AFM or DMS footprint assays to examine DNA triplexes (43,44). Our rescue-rope strategy assisted force-jump assays could examine the triplex forming probability as a function of incubation time, which will be worth to future studies in kinetics. In addition, we have used 20 pN as the testing force to examine the pausing probabilities. The testing force is much higher than the unfolding forces of dsDNA, assuring that the pausing signals come from the formation of telomeric triplexes. Because kinetically short-lived pauses may not survive at 20 pN, the strong testing forces may also underestimate the probability of pauses. Both incubation time and testing forces could be systematically fine-tuned in force-jump assays to reveal the detailed mechanics and dynamics of triplex formation in later research. Furthermore, the G-rich strand containing TTAGGG repeats can form G-quadruplexes, which may result in the low probability of α-shaped structure formation and hence the little chance to observe triplexes.

Imperfect annealing between the G-rich and C-rich strands could be an explanation for the detectable pauses (∼1%) for permutating and deleting mutants of 3′ overhang, as well as the junction mutant. Because our telomeric construct contains a few repeats of TTAGGG in the duplex which allows frame shifting of 6n bp (*n* = 1, 2, 3 or 4), imperfect base pairing in the duplex could generate an ssDNA overhang. A telomeric triplex due to imperfect annealing at the dsDNA/ssDNA junction could thus give detectable pausing signals in our force manipulating protocol.

The rescue-rope strategy expanded the potential of mechanical manipulations on structures formed at freely open ends of DNA. We have revealed rarely formed triplexes of a human telomeric DNA at a physiological pH in a monovalent cation buffer. DNA triplex is one of the unusual conformations formed by telomere, which plays a significant role in chromosome end protection (7,8). Our rescue-rope strategy can mechanically probe a DNA triplex in variable ways, e.g. by unzipping the Watson–Crick duplex, or by breaking reverse Hoogsteen pairs and peeling a TFOs out of the major groove of a dsDNA as we have demonstrated. Mechanical manipulation using our rescue-rope strategy is possible for other ways, e.g. pulling a TFOs out of a triplex in a shearing geometry (25,64–65).

The rescue-rope-strategy assisted single-molecule mechanical manipulation can also be applied to investigate other nucleic acids structures with freely open ends, such as D-loop, T-loop or R-loop (10,66–68). Telomeric G-rich overhang may align with its upstream duplex in parallel regarding the chemically identical strand and invade into the dsDNA by homologous sequence displacement, generating a D-loop with the displaced ssDNA and a T-loop with the folding back dsDNA. To mechanically examine the formation of a D-loop and a T-loop using the rescue-rope strategy, a telomeric DNA construct will be much longer than the current design in this work. Loops during T-loop formation can only happen for long DNA that should overcome the bending energy of dsDNA. The length of telomeric DNA in a rescue-rope construct is scalable by ligation. A rescue-rope setup with an ssDNA chain and a dsDNA hairpin can be applied to directly examine the ssDNA–dsDNA interactions in the process of strand invasion. Mutations in DNA sequence may be necessary to facilitate the strand invasion for D-loops. Similar experimental design can be expanded to investigate R-loops formed by, for example, non-coding RNA TERRA hybridizing with telomeric DNA duplex (69). Proteins, e.g. shelterin complex, may help to make D-loop, T-loop and R-loop frequently happen, which could be probed by multiple manipulation geometries to reveal detailed mechanics and dynamics in telomere protection.

Our single-molecule pulling method may be used to study currently available or to discover new triplex-binding ligands (70,71). For example, we could directly evaluate how ligands affect the mechanical properties and stabilities of triplexes (72–74). We may also investigate the specificity and selectivity of triplex-binding ligands to DNA sequences (75).

## Supplementary Material

gkz464_Supplemental_Files
