# Supplementary material for: The dynamics of forming a triplex in an artificial telomere inferred by DNA mechanics
Source: Nucleic Acids Res. 2019 May 22;47(15):e86. doi: 10.1093/nar/gkz464 (PMC6735771; doi:10.1093/nar/gkz464)
Supplement: gkz464_Supplemental_Files [file gkz464_supplemental_files.pdf]

Supplementary Information

The dynamics of forming a triplex in an artificial telomere inferred by DNA mechanics

Ning Li[1], Junli Wang[1], Kangkang Ma[1], Lin Liang[1], Lipei Mi[2], Wei Huang[1], Xiaofeng Ma[1], Zeyu Wang[1], Wei Zheng[1], Linyan Xu[2], Jun-Hu Chen[3] and Zhongbo Yu[1,*]

[1] State Key Laboratory of Medicinal Chemical Biology, College of Pharmacy, Nankai University, Haihe Education Park, 38 Tongyan Road, Tianjin 300353, China

[2] State Key Laboratory of Precision Measuring Technology and Instruments, Tianjin University, Tianjin 300072, China

[3] National Institute of Parasitic Diseases, Chinese Center for Disease Control and Prevention, WHO Collaborating Center for Tropical Diseases, National Center for International Research on Tropical Diseases, Key Laboratory of Parasite and Vector Biology, Ministry of Health, Shanghai 200025, China

* To whom correspondence should be addressed, Email: zyu@nankai.edu.cn

This Supplementary Information includes:

Table S1. Oligos for constructing the rescue-rope DNA

Table S2. The sequence of the artificial telomere and its mutants

Figure S1. Rescue-rope-strategy validated by AFM and single-molecule magnetic tweezers based on the construct carrying a telomere mutant with 3′ overhang deleted.

Figure S2. Unfolding forces and changes in the extension upon stretching the rescue-rope DNA.

Figure S3. Hopping features in force-extension traces before entirely unfolding the rescue-rope constructs of telomere mutants

Table S1. Oligos for constructing the rescue-rope DNA

Name	Sequence
G-rich strand for artificial telomere	/5AzideN/TT TTA TCA GAT TTT AGG GTT AGG GTT AGG GTT AGG GTT AGG GTT AG**G GTT AGG GTT AGG GTT AG**
G-rich strand for permutation of 3′ overhang	/5AzideN/TT TTA TCA GAT TTT AGG GTT AGG GTT AGG GTT AGG GTT AGG GTT AG **ATGTAGTATGTTGAGTGT**
G-rich strand for deletion of 3′ overhang	/5AzideN/TT TTA TCA GAT TTT AGG GTT AGG GTT AGG GTT AGG GTT AGG GTT AG
G-rich strand for internal loop mutant	/5AzideN/TT TTA TCA GAT TTT AGG G**TA TGT A**GT AGG GTT AGG GTT AGG GTT AGG GTT AGG GTT AGG GTT AG
C-rich strand	CTA ACC CTA ACC CTA ACC CTA ACC CTA ACC CTA AAA TCT GAT AAT T/3AzideN/
G-rich strand for junction mutant	/5AzideN/TTGTCTATCGTACGATGGTTAGGGTTAGGGTTAG **TCATACATGATA**GGTTAGGGTTAGGGTTAG
C-rich strand for junction mutant	**TATCATGTATGA**CTAACCCTAACCCTAACCATCGTACGATA GACTT/3AzideN/
G-rich strand for upstream mutant	/5AzideN/TTGTCTATCGTACGAT**ATTGTGATACTT**GGTTAG GGTTAGGGTTAGGGTTAGGGTTAGGGTTAG
C-rich strand for upstream mutant	CTAACCCTAACCCTAACC**AAGTATCACAAT**ATCGTACGAT AGACTT/3AzideN/
Anchor for C-rich strand	GCATCGgctgaggACGAGAAACG/i5OctdU/AAAATGATAT
Biotin primer	/5Biosg/AGCTGCGTCGTTTGACATCACT
Anchor for G-rich strand	gcatcgCACGAGCATCATACCT/i5OctdU/CCGAGCATTT
Dig primer	/5DigN/ATTCTGGATACCACCACTTA

Forward primer for rescue-rope	GCATCGctcgtgGATTTCGCCAACATCATTCG
Reverse primer for rescue-rope	GCATCGcctcagcTGGCGATTATTATCTTCAGG

Note:

a) /5AzideN/: Azide modification with an NHS Ester at the 5' position in an oligo.

b) /3AzideN/: Azide modification with an NHS Ester at the 3' position in an oligo.

c) /i5OctdU/: 5-Octadiynyl dU with an alkyne group at an internal position in an oligo.

d) /5Biosg/: Biotin-modified oligo at the 5' position.

e) /5DigN/: 5' Digoxigenin (NHS Ester) in an oligo.

Table S2. The sequence of the artificial telomere and its mutants

Name	Sequence
Artificial telomere	5′-TTATCAGATT(TTAGGG)$_5$TTAGGG(TTAGGG)$_2$TTAG<br>3′-AATAGTCTAA(AATCCC)$_5$AATC
Permutation of 3′ overhang	5′-TTATCAGATT(TTAGGG)$_5$TTAG **ATGTAGTATGTTGAGTGT**<br>3′-AATAGTCTAA(AATCCC)$_5$AATC
Deletion of 3′ overhang	5′-TTATCAGATT(TTAGGG)$_5$TTAG<br>3′-AATAGTCTAA(AATCCC)$_5$AATC
Junction mutant	5′-TTGTCTATCGTACGATGG(TTAGGG)$_2$TTAG**TCATACATGATA**GG(TTAGGG)$_2$TTAG<br>3′-TTCAGATAGCATGCTACC(AATCCC)$_2$AATC**AGTATGTACTAT**
Upstream mutant	5′-TTGTCTATCGTACGAT**ATTGTGATACTT**GG(TTAGGG)$_2$TTAGGG(TTAGGG)$_2$TTAG<br>3′-TTCAGATAGCATGCTA**TAACACTATGAA**CC(AATCCC)$_2$AATC
Internal loop mutant	5′-TTATCAGATTTTAGGGT**ATGTAG**TAGGG(TTAGGG)$_2$TTAGGG(TTAGGG)$_2$TTAG<br>3′-AATAGTCTAAAATCCCAATCCCAATCCC(AATCCC)$_2$AATC
AA mutant	5′-TTATCAGATT(AAAGGG)$_5$AAAGGG(AAAGGG)$_2$AAAG<br>3′-AATAGTCTAA(TTTCCC)$_5$TTTC

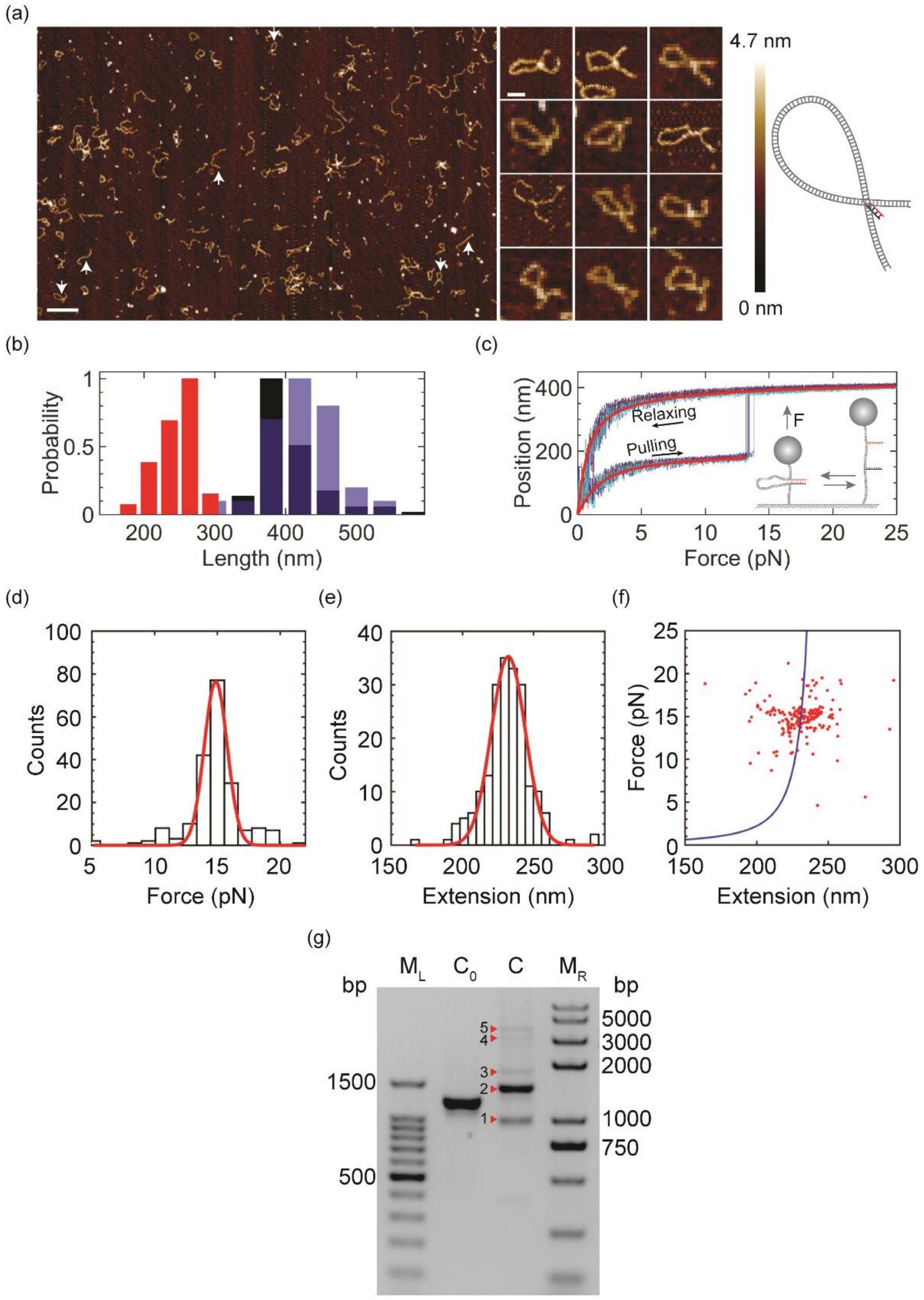

Figure S1. Rescue-rope-strategy validated by AFM, single-molecule magnetic tweezers and agarose gel electrophoresis based on the construct carrying a telomere mutant with 3′ overhang deleted or permutated.

(a) AFM imaging of the rescue-rope DNA. The telomere mutant with a deletion of 3′ overhang ties the rescue-rope DNA into an α-shaped conformation. Upward arrows

and downward arrows point to the linear rescue-rope DNA and α-shaped ones, respectively. The bar is 200 nm for the large panel and 50 nm for small panels. The color bar indicates height values in the AFM images. The cartoon illustrates the α-shaped rescue-rope DNA tied by a telomere mutant. AFM imaging was performed at 23 °C in a buffer containing 10 mM HEPES (pH 7.6), 4 mM $MgCl_2$, and 2 mM $NiCl_2$.

(b) DNA length measured for the α-shaped conformation based on AFM images. The histogram in red indicates the circumference of the closed circle in the α-shaped rescue-rope DNA, 248 nm ± 26 nm (mean ± sd, n = 30). The histogram in blue represents the full length of the α-shaped conformation, 422 nm ± 47 nm (mean ± sd, n = 30). The histogram in black measures the length of linear rescue-rope DNA, 407 nm ± 43 nm (mean ± sd, n =100).

(c) Mechanical manipulation of α-shaped rescue-rope DNA on single-molecule magnetic tweezers. The construct is a telomere mutant with 3′ overhang deleted. Bead positions indicate that the DNA extensions grow as a function of forces, which follows the Worm-Like-Chain model (red line, contour length difference = 233 ± 18 nm, n=9). Five curves are repetitive trajectories of pulling-relaxing from a single molecule. The sudden leaps of bead positions suggest the unfolding of an α-shaped conformation. The cartoon illustrates the setup of a mechanical pulling-relaxing assay, i.e., force-ramp assay. The pulling assays were performed at 23 °C in a buffer containing 20 mM HEPES (pH 7.5), 0.00315% Tween-20(v/v), 0.1 mM EDTA and 100 mM KCl.

(d) Histogram of unfolding forces. Red line indicates a Gaussian fit centered at 15 ± 2 pN (n = 199). The experimental conditions were the same as that in (c).

(e) Histogram of change in bead positions, or DNA extension, before and after the sudden leaps. Red line represents a Gaussian fit centered at 231 ± 16 nm (n = 199). The experimental conditions were the same as that in (c).

(f) A plot showing force vs extension. Red dots are the same data as that in (d-e). The solid blue line was estimated from a WLC model with parameters from (c).

(g) Gel shift assay. The construct is a telomere mutant with 3′ overhang permutated. Lane C contains a rescue-rope construct after treatment of heating and cooling. Lane C_0 has a dsDNA of 1238 bp as a control. M for left (M_L) or right (M_R) markers.

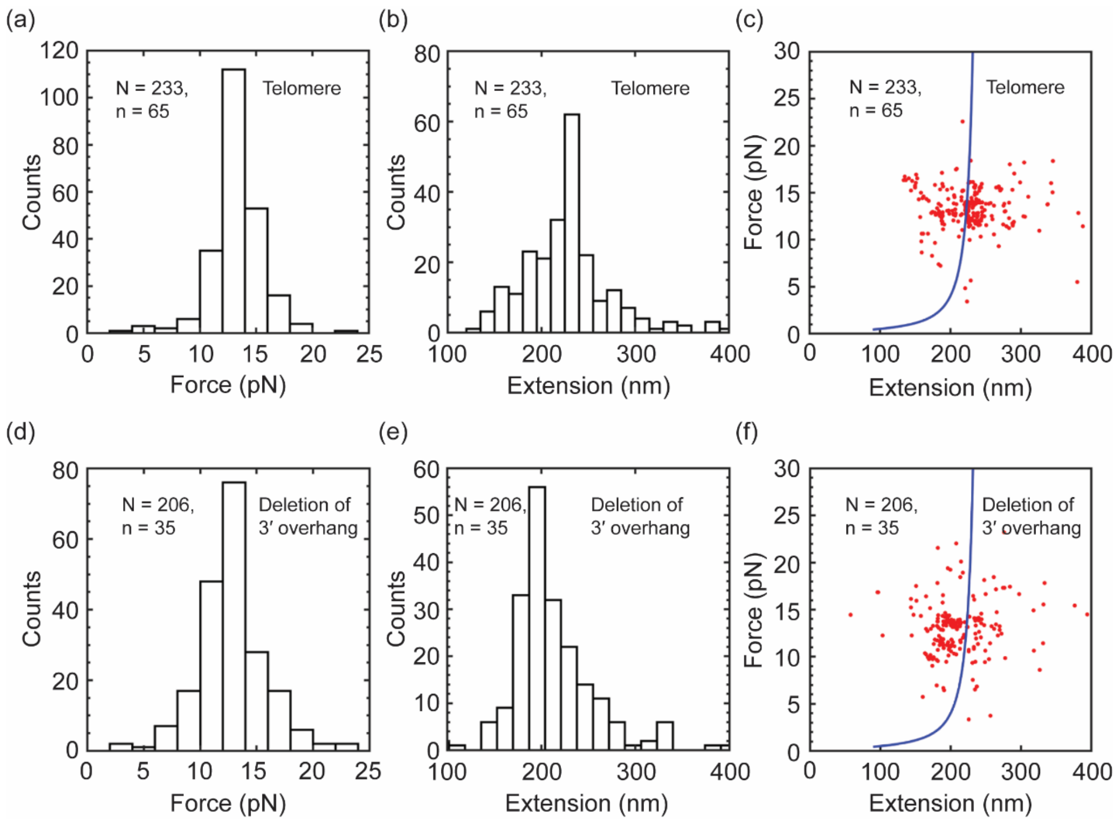

Figure S2. Unfolding forces and changes in the extension upon stretching the rescue-rope DNA.

(a and d) Unfolding-force distributions of the rescue-rope DNA in force-ramp assays revealed by single-molecule magnetic tweezers. The pulling assays were performed at 23 °C in a buffer containing 20 mM HEPES (pH 7.5), 0.00315% Tween-20(v/v), 0.1 mM EDTA and 100 mM NaCl.

(b and e) Changes in the extension before and after the sudden leaps when stretching the rescue-rope DNA. Capital N stands for the total traces of pulling-relaxing in force-ramp assays while lowercase n represents the number of molecules tested. The force loading rate is ± 1 pN/s.

(c and f) Plots showing force vs extension. Red dots in (c) and (f) are the same data as that in (a-b) and (d-e), respectively. The solid blue lines were estimated from a WLC model with parameters from their corresponding pulling-relaxing traces.

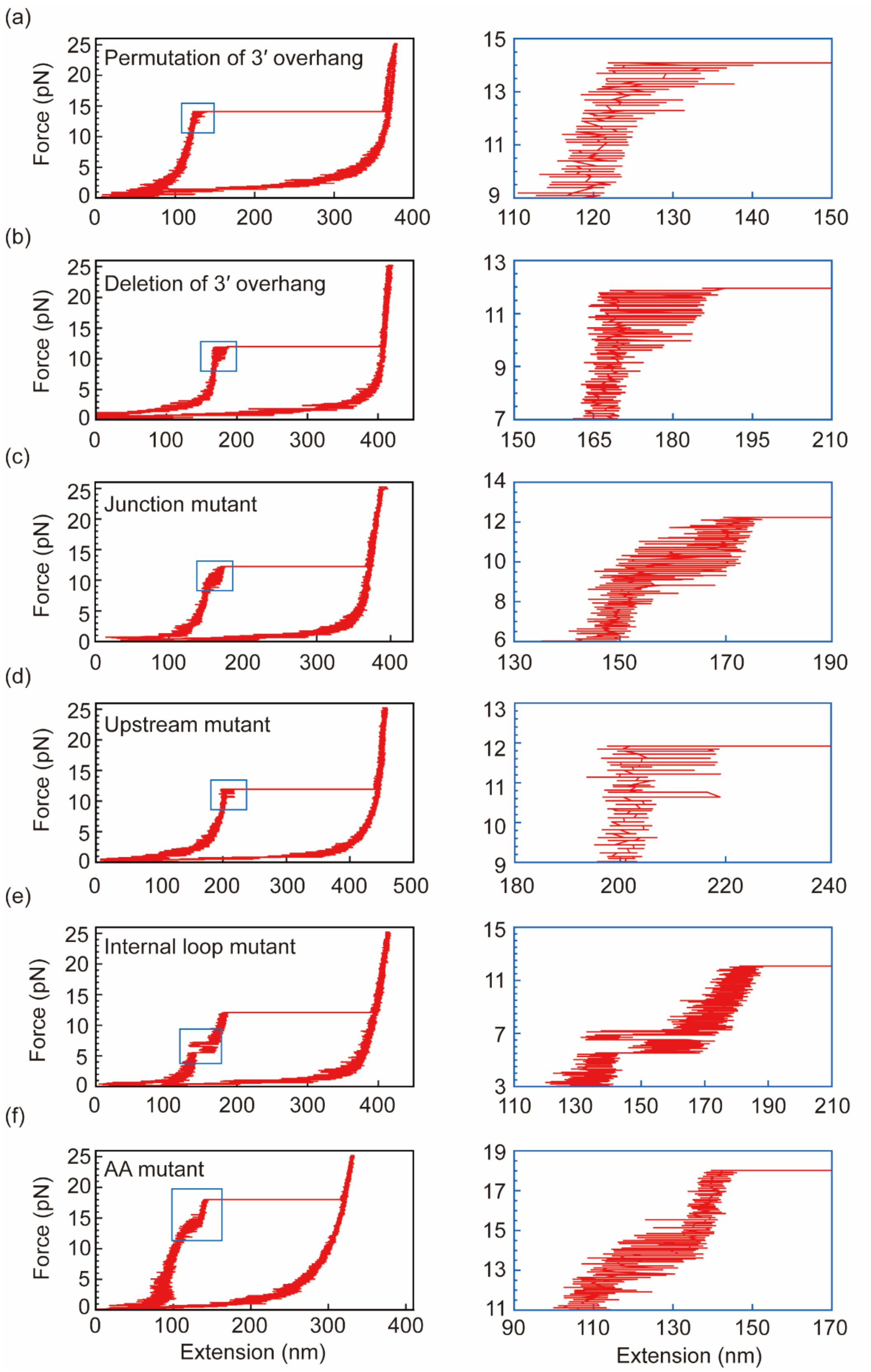

Figure S3. Hopping features in force-extension traces before entirely unfolding the rescue-rope constructs of telomere mutants. The right blue-framed panels show detailed hopping signals for each mutant indicated in its corresponding left panel.
